# Anti-Tuberculosis Therapy-Induced Hepatotoxicity among Ethiopian HIV-Positive and Negative Patients

**DOI:** 10.1371/journal.pone.0001809

**Published:** 2008-03-19

**Authors:** Getnet Yimer, Getachew Aderaye, Wondwossen Amogne, Eyasu Makonnen, Eleni Aklillu, Lars Lindquist, Lawrence Yamuah, Beniyam Feleke, Abraham Aseffa

**Affiliations:** 1 Armauer Hansen Research Institute, Addis Ababa, Ethiopia; 2 Addis Ababa University, Medical Faculty, Addis Ababa, Ethiopia; 3 Department of Clinical Pharmacology, Karolinska Institute, Stockholm, Sweden; 4 Department of Internal Medicine, Infectious Disease Unit, Karolinska Institute, Stockholm, Sweden; 5 St. Peter's TB Specialized Hospital, Addis Ababa, Ethiopia; University of Cape Town, South Africa

## Abstract

**Background:**

To assess and compare the prevalence, severity and prognosis of anti-TB drug induced hepatotoxicity (DIH) in HIV positive and HIV negative tuberculosis (TB) patients in Ethiopia.

**Methodology/Principal Findings:**

In this study, 103 HIV positive and 94 HIV negative TB patients were enrolled. All patients were evaluated for different risk factors and monitored biochemically and clinically for development of DIH. Sub-clinical hepatotoxicity was observed in 17.3% of the patients and 8 out of the 197 (4.1%) developed clinical hepatotoxicity. Seven of the 8 were HIV positive and 2 were positive for HBsAg.

**Conclusions/Significance:**

Sub-clinical hepatotoxicity was significantly associated with HIV co-infection (p = 0.002), concomitant drug intake (p = 0.008), and decrease in CD4 count (p = 0.001). Stepwise restarting of anti TB treatment was also successful in almost all the patients who developed clinical DIH. We therefore conclude that anti-TB DIH is a major problem in HIV-associated TB with a decline in immune status and that there is a need for a regular biochemical and clinical follow up for those patients who are at risk.

## Introduction

The magnitude of tuberculosis (TB) has increased globally since 1990 due to spread of human immunodeficiency virus (HIV) and population growth[1]. It is estimated that *Mycobacterium tuberculosis* has infected one-third of the world's population and 8–9 million new TB cases occur each year. [2]–[7] In populations with high HIV prevalence, TB is the leading cause of morbidity and mortality. [4], [6], [8]

Hepatotoxicity is one of the most important adverse drug reactions associated with anti-tuberculosis drugs that may limit their use.[5] Previous studies showed transient elevations of serum hepatocellular enzymes (e.g. alanine aminotransferase and aspartate aminotransferase) in approximately 10% of patients who received a standard combination chemotherapy including isoniazid and rifampicin, of these 1–2% patients withdrew from the treatment because of severe hepatotoxicity that ultimately led to fulminant hepatitis. [9]

Although the occurrence of drug induced hepatotoxicity (DIH) is difficult to predict, it has been observed that certain patients are at higher risk during the course of anti-TB chemotherapy [10]–[17]. Various studies have suggested that high alcohol intake, older age, slow acetylation status, pre-existing chronic liver disease, chronic viral infection due to hepatitis B and hepatitis C, HIV infection, advanced tuberculosis, Asian ethnicity, female sex, concomitant administration of enzyme-inducers (e.g. barbiturates and anaesthetic agents), inappropriate use of drugs and poor nutritional status increase the risk of anti-tuberculosis drug induced hepatitis. [10]–[17]

It has been suggested that AIDS is significantly associated with development of hepatotoxicity.[18] However, the reason for this association is currently unknown, but may reflect disease-induced alterations in bioactivation or detoxification of reactive metabolites, as well as immunedysregulation. [19] Besides, there has not been any study that showed the association between CD4 count and development of hepatotoxicity among the HIV infected patients. [9]

In this study, we aimed at determining the possible risk factors for development of DIH in both HIV-infected (none of which received ART) and non-infected TB patients from Ethiopia.

## Materials and Methods

In this cohort study Ethiopian TB patients were followed and gave rise to a group of cases of hepatotoxicity and controls (no hepatotoxicity). The study was conducted from August 2004 to March 2005, 103 HIV positive and 94 HIV negative consecutive newly diagnosed male and female adult patients were prospectively evaluated.

Diagnosis of tuberculosis was based on sputum smear, fine needle aspirate (FNA), and clinical and radiological evidence. The criteria for diagnosis was based on the Ethiopian National guideline for diagnosis of tuberculosis.^21^ The number of patients who had smear positive TB were 99, smear negative 39, extra-pulmonary 32, and disseminated 17. Patients were excluded if they had a history of prior treatment for tuberculosis, did not consent for HIV testing, were less than 18 years of age and had clinical evidence of liver damage prior to starting medication. Patients were put on DOTS and regimens with rifampicin, isoniazid, and pyrazinamide (44 patients) or rifampicin, isoniazid, pyrazinamide and either ethambutol (10 patients) or streptomycin (142 patients) were given for 2 months of intensive phase (table 1). During the 6 months of continuation phase all patients were put on rifampicin plus ethambutol.

**Table 1:** Risk factors for development of sub-clinical and clinical hepatotoxicity, Addis Ababa, Ethiopia, August 2004-March 2005

Variable	Status	Sub-clinical DIH		p-value
		Yes	No	
HIV status	Positive	26 (25.2%)	77 (74.8%)	0.002
	Negative	8 (8.5%)	86 (91.5%)	
Type of TB	Pulmonary	25 (17.1%)	121 (82.9%)	0.325
	Extra pulmonary	4 (12.5%)	28 (87.5%)	
	Disseminated	5 (29.4%)	12 (70.6%)	
Anti-TB drugs	SRHZ	27 (19.0%)	115 (81.0%)	0.175
	ERHZ	3 (30.0%)	7 (70.0%)	
	RHZ	4 (9.1%)	40 (90.9%)	
Concomitant drug intake	Yes	16 (29.1%)	39 (70.9%)	0.008
	No	18 (13.0%)	120 (87.0%)	
HbsAg	Positive	3 (21.4%)	11 (78.6%)	0.683
	Negative	31 (17.1%)	150 (82.9%)	
Anti-HCV Antibody	Positive	0 (0%)	4 (100%)	1.000
	Negative	33 (18.6%)	144 (81.8%)	
CD4 count/mm^3^	0–50	8 (53.3%)	7 (46.7%)	0.001
	51–100	8 (25.0%)	24 (75.0%)	
	101–200	4 (18.2%)	18 (81.8%)	
	>200	1 (5.6%)	17 (94.4%)	

For all the patients involved in the study a complete history and physical examination were taken. Laboratory tests done before initiation of anti-TB drugs included; complete and differential blood counts, erythrocyte sedimentation rate (ESR), liver function tests including aspartate aminotransferase (AST), alanine aminotransferase (ALT), direct and total bilurubin and alkaline phosphatase, serological tests for hepatitis B surface antigen (HBsAg) and anti-hepatitis C antibody (anti-HCV). Liver function tests were performed before initiation of anti-TB drugs and late at 1, 2, 4, 6 and 8 weeks after starting DOTS.

Diagnosis of HIV was made based on the National HIV diagnosis algorithm using rapid test kits (Determine, Capillus and Unigold). CD4 count was done for HIV positive patients following the procedure of Flow cytometer after staining the cells within the first week of enrolling participants. FACS COUNT, BECTON DICKINSON, USA is used for counting the CD4 cells.

### Sampling

To answer the question that HIV infection is a risk factor for development of hepatotoxicity in patients taking anti-tuberculosis therapy we set the level of significance and the power of the study at 5% and 80% respectively. A 2.5% and 25% prevalence of anti-TB DIH in HIV negative and HIV positive patients respectively was considered in determining the sample size. The ratio between patients with TB alone and TB/HIV co-infection was taken as 1∶1. Accordingly the required sample size to detect a minimal difference in proportion of patients with hepatotoxicity between the HIV positives and HIV negatives TB patients was 67 per group.

### Diagnosis of anti-tuberculosis DIH (Adopted from the Council for International Organizations of Medical Sciences (CIOMS) scale)

#### 1. Biochemical DIH (Sub-Clinical hepatotoxicity)

Patients were diagnosed to have biochemical hepatotoxicity if there was no apparent cause for the raised liver function tests plus if he/she had one of the following: a rise of five times the upper limit of normal levels (31 U/L) of serum alanine aminotransferase (ALT) and no sign and symptom of DIH.

#### 2. Clinical DIH

Clinical hepatotoxicity was diagnosed if a patient had biochemical hepatotoxicity plus symptoms of DIH including: nausea, vomiting, weakness and jaundice.

### Desensitization protocol for patients who developed clinical DIH

After discontinuing all the anti-tuberculosis therapy, patients were followed by both clinically and biochemical parameters for signs and symptoms of DIH. By the time when all the signs and symptoms of DIH disappeared and the liver function tests dropped down to the normal range, patients were reinitiated on their anti-tuberculosis therapy based on the following protocol. Isoniazid 50 mg per day, followed by isoniazid 100 mg per day, followed by isoniazid 200 mg and rifampicin 300 mg per day, followed by RHZ 1tab and RH 2 tabs per day, followed by RHZ 2 tabs and RH 1 tab per day, followed by RHZE 3 tabs per day, and finally the normal dose for the patient based on the patients weight. Desensitization was done every three days and the dosages of rifampicin and isoniazid in RH were 150 mg and 75 mg, rifampicin, isoniazid and pyrazinamide in RHZ were 150 mg, 75 mg, and 400 mg and that of rifampicin, isoniazid, pyrazinamide and ethambutol in the RHZE were 150 mg, 75 mg, 400 mg, and 275 mg respectively. The intensive phase was also prolonged by considering as if the patient was not on treatment during the discontinuation and desensitization period. This desensitization protocol was adopted from the normal hospital practice for management of DIH in the study site.

### Ethical consideration

The study protocol was approved by two institutional IRBs and one National Ethical Review Committee which gives the final decision at the national level. Therefore, ethical approval was obtained from: IRB of Armauer Hansen research Institute, FRPC (Faculty research and publication committee) of Faculty Medicine Addis Ababa University, and the National Health Science and Technology Council and Ethics Review Committee. Written informed consent for HIV testing and participation in the study was obtained from all participants prior to the commencement of the study. Opportunistic infections were treated free of charge. HIV positive patients were eventually linked to HIV patients care and support center. Due to the unavailability of antiretroviral drugs nationally at the time of the study patients were not provided ART.

### Statistical analysis

Data were double entered and checked. HIV positive and negative TB patients were compared and SPSS version 11.0 was used for analysis of the different risk factors for development of Hepatotoxicity. Chi-square, Fishers exact tests and multivariate analysis were used to test for the level of significance. Multiple regression analysis was used to see the effect of different confounding factors. Clinical and biochemical hepatotoxicity were the dependant variables whereas age, sex, HBsAg, concomitant drug intake, HIV status, body mass index, CD4 count and anti-HCV antibody were the independent variables. OR and P-values are used to see the significant risk factors. Variables with p<0.05 were considered potential predictors of DIH.

## Results

### Patients

Out of the 197 study participants 105 (52.8%) were male and 92 (47.2%) were female; the median age of the patients was 26 years (range, 18 to 67 years). Out of these patients, 103 (52.3%) patients were HIV positive and 94 (47.7%) patients were negative. CD4 count was done for 87 of the 103 HIV positive patients and the distribution is shown in Table 1. Eighty percent of the patients had CD4 count less than 200 cells/mm^3^.Fourteen (7.2%) were positive for HBsAg and only 4 (2.2 %) were positive for anti-HCV.

Among the 195 patients 146 (74.9%) had pulmonary tuberculosis of whom 107 (73.3%) had smear positive and 39 (26.7%) had smear negative TB. Thirty two (16.4%) of the patients had extra pulmonary TB and 17 (8.7%) had disseminated TB (Table 2).

**Table 2:** Frequency distribution of demographic and clinical variables, Addis Ababa, Ethiopia, August 2004-March 2005

Variables	Status	Number of patients (%)	Total number patients
Age (years)	<35	131 (67.2)	195
	≥35	64 (32.8)	
Sex	Male	105 (53.3)	197
	Female	92 (46.7)	
Body mass index	<18.5	108 (58.7)	184
	18.5–24.9	71 (38.6)	
	25–29.9	5 (2.7)	
History of Jaundice	Yes	14 (7.2)	194
	No	180 (92.8)	
History of blood transfusion	Yes	5 (2.6)	196
	No	191 (97.4)	
History of chronic illness	Yes	5 (2.6)	196
	No	191 (97.4)	
Traditional medicine intake	Yes	35 (18.2)	192
	No	157 (81.8)	
Alcohol intake	Yes	74 (38.1)	194
	No	120 (61.9)	
HIV status	Positive	103 (52.3)	197
	Negative	94 (47.7)	
Type of TB	Pulmonary	146 (74.9)	195
	Extra pulmonary	32 (16.4)	
	Disseminated	17 (8.7)	
Concomitant drug intake	Yes	55 (28.5)	193
	No	138 (71.5)	
HBsAg	Positive	14 (7.2)	195
	Negative	181 (92.8)	
Anti-HCV Ab	Positive	4 (2.2)	181
	Negative	177 (97.8)	
CD4 count/mm^3^	0–50	15 (17.2)	87
	51–100	32 (36.8)	
	101–200	22 (25.3)	
	>200	18 (20.7)	

Only 55 (27.9%) patients from the 197 took other drugs concomitantly and these drugs were: Vitamin B_6_ (7 patients), codeine phosphate (5 patients), promethazine (4 patients) amoxicillin (13 patients), antiacid syrup (13), albendazole (3 patients), tetracycline (2 patients), phenobarbitone (2 patients), ampicillin and gentamincine (2 patients), phenobarbitone plus antiacid syrup (1 patient), ketoconazole (1 patient), and trimethoprim/sulfamethoxazole (2 patients).

The Body Mass Index (BMI) of the HIV positive patients was <18.5 for 61 (59.2%) whereas BMI for the HIV negative patients was <18.5 for 47 (50.0%) and the differences were not significant between the 2 groups of patients (p>0.05)(Table 2). However, comparison of BMI in patients with and with out DIH was not done as the number of patients with DIH is very small.

### Association of sub-clinical hepatotoxicity with demographic and clinical variables

Thirty four (17.3%) study participants had sub-clinical hepatotoxicity of which 8 (4.1%) developed clinical hepatotoxicity with all of them having jaundice and necessitated discontinuation of their anti-tuberculosis therapy. Out of the 34 patients 14 (41.2%) were males and 20 (58.8%) were females. Twenty six (76.5%) were HIV positive and 8 (23.5%) were negative. Twenty two (66.7%) patients were <35 years of age and the mean time for the development of sub-clinical DIH was 2.8 weeks (Range 1–8 weeks). The mean duration of stay of the elevated ALT was 2.2 weeks (Range 1–7 weeks).

Multivariate and bivariate analyses were performed to see the association between different variables and development of clinical and biochemical hepatotoxicity. The effect of confounders such as age, sex, HBsAg, concomitant drug intake, HIV status, body mass index, CD4 count and anti-HCV antibody was controlled. The results of the analyses showed that the odds of developing biochemical hepatotoxicity in patients taking other drugs concomitantly was 2.7 with a 95% CI of 1.2–5.8. The odds of developing biochemical hepatotoxicity in HIV positive patients was 3.6 with a 95% CI of 1.5–8.5 as compared to that of HIV negative patients (Table 1, 3 and 4).

**Table 3:** Unadjusted analyses of the association between sub-clinical hepatotoxicity with different demographic and clinical variables, Addis Ababa, August 2004-March 2005

Variable	Status	OR (95% CI )
HIV status	Positive	3.6 (1.5–8.5)
	Negative	1.0
Sex	Female	1.8 (.8–3.8)
	Male	1.0
Age	>35	1.1 (.48–2.4)
	<35	1.0
BMI	≥18.5	0.7 (.3–1.5)
	<18.5	1.0
Concomitant drug intake	No	0.4 (.2–.9)
	Yes	1.0
HbsAg	Negative	0.7 (.2–2.5)
	Positive	1.0
Anti-HCV Ab	Negative	1.0 (0.9–1.0)
	Positive	1.0
CD4 count/mm3	0–50	21.3 (2.2–204.9)
	51–100	5.2 (0.6–46.0)
	101–200	4.3 (0.4–42.4)
	>200	1.0

**Table 4:** Multivariate analyses of an association between sub-clinical hepatotoxicity with different clinical variables, Addis Ababa, August 2004–March 2005

Variable	Status	OR (95% CI )
HIV status	Positive	3.6 (1.5–8.5)
	Negative	1.0
Concomitant drug intake	Yes	2.7 (1.2–5.8)
	No	1.0
HbsAg	Positive	1.3 (.35–5.0)
	Negative	1.0
Anti-HCV Antibody	Positive	307.5 (0.0–1.2)
	Negative	1.0
CD4 count/mm3	0–50	20.5 (2.1–195.6)
	51–100	5.9 (0.6–52.2)
	101–200	4.2 (0.4–41.7)
	>200	1.0

Three (21.4%) of the HBsAg positive and 31 (17.1%) of the HBsAg negative study participants developed sub-clinical hepatotoxicity whereas none of the 4 anti-HCV positive patients had hepatotoxicity and no patient was positive for concomitant HBsAg and anti-HCV Ab.

CD4 count was done for the HIV positive patients and multivariate analysis to see the association with development of sub-clinical hepatotoxicity showed that among those patients with different CD4 count when compared with those with >200/mm^3^ the odds ratio is 20.5 with a 95% CI (2.1–195.6) for those with 0–50/mm^3^, 5.9 with a 95% CI (0.6–52.2) for those with 51–100/mm^3^, and 4.2 with a 95% CI (0.4–41.7) for those with 101–200/mm^3^ (Table 1, 3 and 4). This suggests that as the CD4 count decreases the risk of developing hepatotoxicity also increases.

## Discussion

The proportion of patients with anti-tuberculosis therapy induced hepatotoxicity in general has not been studied that often in African patients. In this study therefore, we studied this common clinical problem among Ethiopian patients. To effectively study this problem we have excluded patients previously treated with anti-tuberculosis drugs, critically sick patients as assessed by level of consciousness and concomitant illnesses, and patients with baseline liver function test (LFT) values above the normal range.

The proportion of DIH in HIV positive and HIV negative patients was statistically significant where HIV positive patients are at a higher risk than HIV negative ones. Similar findings have been observed in other studies. [13], [20], [21] This may be partly explained by the different drugs used by these patients for the treatment of AIDS-related opportunistic infections before the diagnosis of TB was made. Even if the number of patients in the different groups is small we have also observed that development of hepatotoxicity had a statistically significant association with decrement in the immune status of the patients as measured by the CD4 count. This phenomenon was not shown in previous studies and may suggest the presence of an immunologic mechanism for the development of anti-TB DIH although the exact mechanism has not yet been clearly elucidated. [22] The other possible explanation for this could be, since patients with low CD4 count are more prone in acquiring opportunistic infections, this might necessitate consumption of different drugs leading to sub clinical liver damage and thereby increase susceptibility for hepatotoxicity while taking anti TB. [8], [23], [24]

Unlike previous studies which showed that patients in the older age group are at increased risk for development of hepatotoxicity, [13], [20], [25] in this study, such a finding was not observed and this could be due to a larger proportion of study participants being in the age group below 35 years which will make comparison difficult. However, there are also studies which support that age is not a risk factor for development of hepatotoxicity in patients taking anti-tuberculosis therapy. [25]–[27]

Malnutrition as a risk factor for DIH was suggested by previous studies. [12], [25] However, in this study we did not see any significant association between malnutrition as measured by BMI and DIH. Perhaps this may be because of the larger proportion of study participants having a lower BMI making comparison difficult. Moreover, similar findings were seen in some other studies. [22], [26]

Being alcoholic is one of the well established risk factors for DIH. [12], [21], [22], [26] In our study however, we did not see this association. The reason for this is that even if 74 patients replied that they have history of alcohol intake it is possible that the levels of alcohol consumption were not high enough to contribute to hepatotoxicity.

We have also observed that patients who took drugs concomitantly with the anti-tuberculosis therapy were at risk for the development of hepatotoxicity. This could be because of the synergistic hepatotoxic effect caused by the other drugs. Similar finding was observed while combining different drugs. [21], [28]

Unlike other studies no association between Anti-HCV and HBsAg positivity and development of hepatotoxicity was observed. [13], [26], [29], [30] This may be attributed to the small number of patients who are positive for Anti-HCV antibody and HBsAg in this study.

This study was conducted at the time were there was no free antiretroviral drug provision currently however, ART is being given for free to all those who are in need. The result of this study might have been affected if there was concurrent treatment with ART. Findings of this study therefore, would act as a background for studies that have aimed at identifying risk factors for DIH while patients take ART and anti-TB drugs concomitantly.

In general we have tried to explore the different risk factors for the development of anti-tuberculosis DIH in newly diagnosed TB patients from Ethiopia. We recommend that patients with HIV infection, low CD4 count and those on other concomitant drugs have initial screening with liver function test and closely monitored with subsequent tests during treatment.

We also recommend that further studies should be conducted to explore the detail mechanism as to why this identified risk factors contribute for development of hepatotoxicity and also further asses those risk factors that are not addresses in this study in detailed.

### Limitations of the study

Since the main objective of this study was to asses the effect of HIV infection on development of hepatotoxicity. The sample size was therefore calculated considering HIV positive and HIV negative patients. As a result it was difficult to make strong association on different parameters such as association of hepatotoxicity with HbsAg, Anti-HCV Antibody and CD4 count. Moreover, since the majority of our study participants were underweight it was difficult to see the association between BMI and hepatotoxicity. The other limitation of the study was representatives. Since the study was conducted only in one health institution which was also located in one city there might be a problem of representing the whole country with regard to economic status and ethnic group.
